# Cystatin C predicts long term mortality better than creatinine in a nationwide study of intensive care patients

**DOI:** 10.1038/s41598-021-85370-8

**Published:** 2021-03-15

**Authors:** Johanna Helmersson-Karlqvist, Miklos Lipcsey, Johan Ärnlöv, Max Bell, Bo Ravn, Alain Dardashti, Anders Larsson

**Affiliations:** 1 Department of Medical Sciences/Clinical Chemistry, Uppsala University, 751 85 Uppsala, Sweden; 2 Hedenstierna Laboratory, CIRRUS, Department of Surgical Sciences, Anesthesiology and Intensive Care, Uppsala University, Uppsala, Sweden; 3 Division of Family Medicine and Primary Care, Department of Neurobiology, Care Sciences and Society (NVS), Karolinska Institutet, Huddinge, Sweden; 4 School of Health and Social Studies, Dalarna University, Falun, Sweden; 5 Department of Anesthesiology, Surgical Services and Intensive Care, Karolinska University Hospital, Stockholm, Sweden; 6 Department of Physiology and Pharmacology, Karolinska Institutet, Stockholm, Sweden; 7 Department of Cardiothoracic Surgery, Anesthesia and Intensive Care, Skane University Hospital, Lund, Sweden

**Keywords:** Kidney diseases, Risk factors, Prognostic markers

## Abstract

Decreased glomerular filtration rate (GFR) is linked to poor survival. The predictive value of creatinine estimated GFR (eGFR) and cystatin C eGFR in critically ill patients may differ substantially, but has been less studied. This study compares long-term mortality risk prediction by eGFR using a creatinine equation (CKD-EPI), a cystatin C equation (CAPA) and a combined creatinine/cystatin C equation (CKD-EPI), in 22,488 patients treated in intensive care at three University Hospitals in Sweden, between 2004 and 2015. Patients were analysed for both creatinine and cystatin C on the same blood sample tube at admission, using accredited laboratory methods. During follow-up (median 5.1 years) 8401 (37%) patients died. Reduced eGFR was significantly associated with death by all eGFR-equations in Cox regression models. However, patients reclassified to a lower GFR-category by using the cystatin C-based equation, as compared to the creatinine-based equation, had significantly higher mortality risk compared to the referent patients not reclassified. The cystatin C equation increased C-statistics for death prediction (*p* < 0.001 vs. creatinine, *p* = 0.013 vs. combined equation). In conclusion, this data favours the sole cystatin C equation rather than the creatinine or combined equations when estimating GFR for risk prediction purposes in critically ill patients.

## Introduction

Glomerular filtration rate (GFR) is the best routinely available estimate for kidney function and essential for detection and management of both acute kidney injury (AKI) and chronic kidney disease (CKD). Loss of kidney function by decreased estimated glomerular filtration rate (eGFR) is associated with poor survival^1^. Creatinine is the most frequently used biomarker for eGFR. Yet, creatinine may vary with factors not related to kidney function per se such as muscle mass, gender, ethnicity, and dietary factors^2,3^. Cystatin C is an alternative biomarker for eGFR-estimation which does not depend on muscle mass and thus fairly constant with age and gender^4,5^. Still, creatinine is the most frequently used estimate of eGFR in critically ill patients^6,7^. Patients in intensive care are often bedfast and may have loss of muscle mass and altered distribution volumes due to severe illness. An ongoing loss of muscle mass and low protein intake may possibly lead to a decrease in creatinine in plasma, leading to potential risk of eGFR overestimation^8^. It may therefore be hypothesized that creatinine is a less informative biomarker in the estimation of eGFR than cystatin C. Cystatin C may on the other hand be influenced by cortisol, obesity and other traditional risk factors or possibly inflammation^9–13^, which varies in critically ill. The performance of creatinine and cystatin C in estimating long-term mortality is less studied in critically ill patients.

The aim of this study was to study the predictive value of creatinine and cystatin C in critically ill patients by investigating if cystatin C improves the association between eGFR and mortality, compared to creatinine, in this particular patient group using reclassification and model discrimination with C-statistics. The study includes a large number of intensive care patients from three Swedish University Hospitals and compares eGFR calculated by creatinine and cystatin C, respectively, and mortality risk at a median follow-up of 5 years.

## Results

### Baseline characteristics and mortality risks in eGFR subgroups

Out of the 22,488 included patients, 10,392 (46%) were admitted to a general intensive care unit, 2597 (11%) to a neurosurgical intensive care unit, 5132 (23%) to a cardiothoracic intensive care unit and 4367 (19%) were admitted to a coronary care unit. Table 1 shows the baseline characteristics of the study participants. Cardiovascular diseases, including hypertension and diabetes mellitus, infections, and trauma were the most common diagnoses among the included patients.

**Table 1:** Basic characteristics of all study participants and in the subgroup general ICU in median (interquartile interval).

	All	General ICU
Number	22,488	10,392
Age	65 (53–74)	62 (45–73)
Female gender (%)	36	40
Mortality within 7 days (%)	7	11
Mortality within 14 days (%)	8	13
Mortality within 30 days (%)	11	16
Mortality within 90 days (%)	14	21
Total mortality during follow-up (%)	37	49
Plasma Creatinine, µmol/L	81 (64–112)	85 (63–134)
Plasma Cystatin C, mg/L	1.06 (0.81–1.52)	1.07 (0.75–1.75)
eGFR crea, ml/min/1,73 m^2^	81 (53–98)	79 (42–103)
eGFR comb, ml/min/1,73 m^2^	75 (47–99)	73 (38–106)
eGFR cyst C, ml/min/1,73 m^2^	68 (44–95)	68 (37–105)
**Discharge diagnoses**		
Infectious disease (%)	24.8	36.4
Diabetes mellitus (%)	15.7	13.4
Hypertension (%)	29.8	20.7
Cardiovascular disease (%)	38.3	19.4
Cerebrovascular disease (%)	10.5	6.1
Liver and biliary tract diseases (%)	3.7	6.8
Kidney diseases (%)	8.7	13.4
Trauma (%)	13.0	20.9
Intoxications (%)	0.7	1.3
Charlson Comorbidity Index (95% CI)	1.56 (1.54–1.59)	1.85 (1.80–1.89)

**Figure 1:**
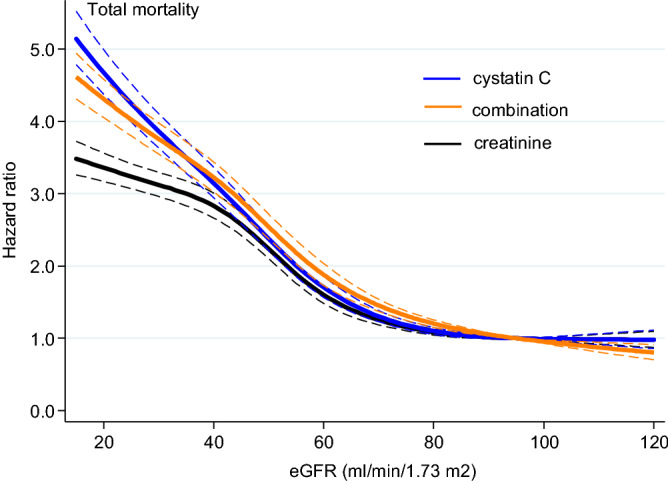
The hazard ratios and 95% confidence intervals (in thin dotted lines) for mortality by each equation from univariate Cox proportional-hazard models are shown in regression spline curves. The reference point is set to 95 ml/min/1.73 m^2^.

During follow-up (median [interquartile interval] 5.1 [2.3–7.1] years, corresponding to 106,036 person-years in total) 8401 (37%) participants died. Hazard ratios with 95% confidence intervals for mortality of all causes for each equation versus the reference point at 95 ml/min/1.73 m^2^ are shown in cubic spline curves (Fig. 1). At eGFR values below 30–40 ml/min/1.73 m^2^ the mortality risk was significantly higher with an equation containing cystatin C, alone or in combination with creatinine, compared to the equation with only creatinine. Harrell´s C statistics (95% confidence interval) for Cox regression models predicting mortality was 0.640 (0.631–0.649) for eGFR_Cr_, 0.664 (0.655–0.673, *P* < 0.001 vs. eGFR_Cr_) for eGFR_Comb_ and 0.667 (0.658–0.676, *P* < 0.001 vs. eGFR_Cr_ and *P* = 0.013 vs eGFR_Comb_) for eGFR_Cyst_. Thus, the equation with only Cystatin C significantly increased the C-statistics for the prediction of death as compared to the equations with Creatinine, both sole and combined.

Incidence rates for mortality according to the eGFR categories > 60, 60–30, 30–20 and < 20 mL/min/1.73m^2^, defined by respective equation, are presented in Table 2. Overall, decreasing eGFR, irrespective of equation, significantly associated with mortality risk (Table 2, Fig. 1).

**Table 2:** All-cause mortality estimates by different eGFR equations using Cox regression models.

	ml/min/1.73 m^2^	N at risk/N of events	IR (95% CI)	HR (95% CI)
eGFR Creatinine	≥ 60	15,749/4404	5.4 (5.2–5.6)	Ref.
	60–30	4381/2384	14.2 (13.6–14.7)	2.4 (2.3–2.5)
	30–20	1112/744	20.6 (19.2–22.2)	3.4 (3.2–3.7)
	< 20	1246/869	21.8 (20.4–23.3)	3.6 (3.3–3.8)
eGFR Cystatin C	≥ 60	13,271/3200	4.5 (4.3–4.7)	Ref.
	60–30	6103/2974	11.7 (11.2–12.1)	2.4 (2.3–2.6)
	30–20	1526/1027	21.2 (20.0–22.6)	4.1 (3.8–4.4)
	< 20	1588/1200	27.3 (25.8–28.9)	5.0 (4.7–5.4)
eGFR Combined	≥ 60	14,556/3734	4.8 (4.7–5.0)	Ref.
	60–30	5069/2649	13.2 (12.7–13.7)	2.5 (2.4–2.6)
	30–20	1347/902	20.6 (19.3–21.9)	3.7 (3.5–4.0)
	< 20	1516/1116	25.4 (23.9–26.9)	4.4 (4.2–4.8)

*IR* incidence rate, *HR* hazard ratio.

### Comparing eGFR_Cyst_ with eGFR_Cr_ using reclassification analysis

Overall, patients reclassified to a lower GFR-category by using the cystatin C-based equation, as compared to the creatinine-based equation, had significantly higher mortality risk (Table 3) compared to the referent patients not reclassified. Conversely, patients reclassified to a higher GFR-category by the cystatin C-based formula, as compared to the creatinine-based equation, had significantly lower mortality risk. These associations were also seen in the subgroup of patients at general ICU. Adjusting for age, gender and cci did in some comparisons give weaker and no longer significant associations. However, in reclassification to lower category by cystatin C almost all associations were still present after adjustments. Altogether, the calculated NRI for the cystatin C equation compared to creatinine equation was 0.13, *P* < 0.001, indicating improved reclassification by the cystatin C equation.

**Table 3:** Mortality risk when classified according to eGFR recalculated with cystatin C, as compared to creatinine, in the whole study population (all) and in general ICU.

	Evaluated (N)	Reclassified to higher eGFR		Not reclassified		Reclassified to lower eGFR	
		N (%)	HR (95% CI)	N (%)	HR (95% CI)	N (%)	HR (95% CI)
**All**							
				2960 events		1444 events	
eGFR ≥ 60	15,749	NA	NA	12,658 (80)	ref	3091 (20)	2.37 (2.23–2.53)
eGFR ≥ 60, adj	15,749	NA	NA	12,658 (80)	ref	3091 (20)	1.51 (1.41–1.61)
		228 events		1452 events		704 events	
eGFR 60–30	4381	588 (13)	0.69 (0.60–0.79)	2822 (64)	ref	971 (22)	1.80 (1.64–1.97)
eGFR 60–30, adj	4381	588 (13)	0.84 (0.73–0.97)	2822 (64)	ref	971 (22)	1.62 (1.48–1.77)
		132 events		293 events		319 events	
eGFR 30–20	1112	257 (23)	0.71 (0.58–0.88)	488 (40)	ref	406 (37)	1.40 (1.20–1.64)
eGFR 30–20, adj	1112	257 (23)	0.92 (0.74–1.13)	488 (40)	ref	406 (37)	1.46 (1.24–1.71)
		161 events		708 events			
eGFR ≤ 20	1246	276 (22)	0.70 (0.59–0.83)	970 (78)	ref	NA	NA
eGFR ≤ 20, adj	1246	276 (22)	0.87 (0.73–1.03)	970 (78)	ref	NA	NA
**General ICU**							
				1720 events		716 events	
eGFR ≥ 60	6523	NA	NA	5398 (83)	ref	1125 (17)	2.74 (2.52–3.00)
eGFR ≥ 60, adj	6523	NA	NA	5398 (83)	ref	1125 (17)	1.63 (1.49–1.79)
		183 events		847 events		405 events	
eGFR 60–30	2144	397 (19)	0.56 (0.48–0.66)	1243 (58)	ref	504 (24)	1.44 (1.28–1.62)
eGFR 60–30, adj	2144	397 (19)	0.68 (0.58–0.80)	1243 (58)	ref	504 (24)	1.39 (1.23–1.56)
		111 events		202 events		222 events	
eGFR 30–20	740	203 (27)	0.63 (0.50–0.79)	272 (37)	ref	265 (36)	1.25 (1.03–1.51)
eGFR 30–20, adj	740	203 (27)	0.81 (0.64–1.02)	272 (37)	ref	265 (36)	1.34 (1.10–1.62)
		147 events		553 events			
eGFR ≤ 20	985	245 (25)	0.69 (0.58–0.83)	740 (75)	ref	NA	NA
eGFR ≤ 20, adj	985	245 (25)	0.84 (0.70–1.01)	740 (75)	ref	NA	NA

Adj = adjusted for age, gender and Charlson Comorbidity Index. HR = hazard ratio.

### Comparing eGFR_Cyst_ with eGFR_Comb_ using reclassification analysis

Generally, patients reclassified to a lower GFR-category by using the cystatin C-based equation, as compared to the combined equation, had significantly higher mortality risk (Table 4) compared to the referent patients not reclassified. Also, patients reclassified to a higher GFR-category by the cystatin C-based formula, as compared to the combined equation, had significantly lower mortality risk. Adjusting for age, gender and cci did in some comparisons give weaker and no longer significant associations, particularly in the subgroup general ICU. Altogether, the calculated NRI for the cystatin C equation compared to the combined equation was 0.04, *P* < 0.001, indicating an improved reclassification.

**Table 4:** Mortality risk when classified according to eGFR recalculated with cystatin C, as compared to the combination formula, in the whole study population (all) and in general ICU.

	Evaluated (N)	Reclassified to higher eGFR		Not reclassified		Reclassified to lower eGFR	
		N (%)	HR (95% CI)	N (%)	HR (95% CI)	N (%)	HR (95% CI)
**All**							
				3072 events		662 events	
eGFR ≥ 60	14,556	NA	NA	12,977 (89)	ref	1579 (11)	1.99 (1.83–2.17)
eGFR ≥ 60, adj	14,556	NA	NA	12,977 (89)	ref	1579 (11)	1.38 (1.27–1.51)
		126 events		2169 events		354 events	
eGFR 60–30	5069	292 (6)	0.81 (0.68–0.97)	4275 (84)	ref	502 (10)	1.82 (1.62–2.03)
eGFR 60–30, adj	5069	292 (6)	0.94 (0.78–1.12)	4275 (84)	ref	502 (10)	1.67 (1.49–1.87)
		138 events		585 events		178 events	
eGFR 30–20	1344	236 (18)	0.84 (0.70–1.00)	875 (65)	ref	233 (17)	1.30 (1.10–1.53)
eGFR 30–20, adj	1344	236 (18)	0.96 (0.80–1.16)	875 (65)	ref	233 (17)	1.27 (1.07–1.50)
		113 events		1003 events			
eGFR ≤ 20	1516	185 (12)	0.70 (0.57–0.84)	1331 (88)	ref	NA	NA
eGFR ≤ 20, adj	1516	185 (12)	0.81 (0.66–0.98)	1331 (88)	ref	NA	NA
**General ICU**							
				1812 events		316 events	
eGFR ≥ 60	6150	NA	NA	5614 (91)	ref	536 (9)	2.32 (2.06–2.62)
eGFR ≥ 60, adj	6150	NA	NA	5614 (91)	ref	536 (9)	1.48 (1.31–1.67)
		100 events		1211 events		208 events	
eGFR 60–30	2290	202 (9)	0.64 (0.52–0.78)	1818 (79)	ref	270 (12)	1.45 (1.25–1.68)
eGFR 60–30, adj	2290	202 (9)	0.71 (0.58–0.87)	1818 (79)	ref	270 (12)	1.42 (1.23–1.65)
		113 events		375 events		102 events	
eGFR 30–20	805	177 (22)	0.79 (0.64–0.98)	501 (62)	ref	127 (16)	1.15 (0.92–1.43)
eGFR 30–20, adj	805	177 (22)	0.90 (0.73–1.12)	501 (62)	ref	127 (16)	1.31 (1.05–1.64)
		102 events		766 events			
eGFR ≤ 20	1145	158 (14)	0.71 (0.58–0.88)	987 (86)	ref	NA	NA
eGFR ≤ 20, adj	1145	158 (14)	0.82 (0.67–1.01)	987 (86)	ref	NA	NA

Adj = adjusted for age, gender and Charlson Comorbidity Index.

### Comparing eGFR_Comb_ with eGFR_Cr_ using reclassification analysis

Patients reclassified to a lower GFR-category by using the combined equation, as compared to the sole creatinine equation, had significantly higher mortality risk (Table 5) compared to the referent patients not reclassified. Patients reclassified to a higher GFR-category by the combined formula, as compared to the sole creatinine equation, had significantly lower mortality risk. Adjusting for age, gender and CCI did not substantially alter the associations. Altogether, the calculated NRI for the combined equation compared to the sole creatinine equation was 0.11, *P* < 0.001, indicating improved reclassification.

**Table 5:** Mortality risk when classified according to eGFR recalculated with a combination formula, as compared to creatinine, in the whole study population (all) and in general ICU.

	Evaluated (N)	Reclassified to higher eGFR		Not reclassified		Reclassified to lower eGFR	
		N (%)	HR (95% CI)	N (%)	HR (95% CI)	N (%)	HR (95% CI)
**All**							
				3616 events		788 events	
eGFR ≥ 60	15,749	NA	NA	14,225 (90)	ref	1524 (10)	2.51 (2.33–2.72)
eGFR ≥ 60, adj	15,749	NA	NA	14,225 (90)	ref	1524 (10)	1.52 (1.40–1.65)
		118 events		1803 events		463 events	
eGFR 60–30	4381	331 (8)	0.59 (0.49–0.71)	3417 (78)	ref	633 (14)	1.75 (1.57–1.94)
eGFR 60–30, adj	4381	331 (8)	0.76 (0.63–0.92)	3417 (78)	ref	633 (14)	1.56 (1.41–1.73)
		55 events		411 events		278 events	
eGFR 30–20	1112	120 (11)	0.62 (0.47–0.82)	640 (58)	ref	351 (32)	1.51 (1.30–1.76)
eGFR 30–20, adj	1112	120 (11)	0.86 (0.65–1.15)	640 (58)	ref	351 (32)	1.49 (1.27–1.73)
		59 events		810 events			
eGFR ≤ 20	1246	112 (9)	0.62 (0.48–0.81)	1139 (91)	ref	NA	NA
eGFR ≤ 20, adj	1246	112 (9)	0.75 (0.58–0.98)	1139 (91)	ref	NA	NA
**General ICU**							
				2035 events		401 events	
eGFR ≥ 60	6523	NA	NA	5933 (91)	ref	590 (9)	2.88 (2.59–3.22)
eGFR ≥ 60, adj	6523	NA	NA	5933 (91)	ref	590 (9)	1.66 (1.48–1.85)
		93 events		1069 events		273 events	
eGFR 60–30	2144	217 (10)	0.52 (0.42–0.65)	1593 (74)	ref	334 (16)	1.49 (1.30–1.70)
eGFR 60–30, adj	2144	217 (10)	0.68 (0.55–0.84)	1593 (74)	ref	334 (16)	1.42 (1.25–1.63)
		46 events		291 events		198 events	
eGFR 30–20	739	99 (13)	0.54 (0.39–0.73)	405 (55)	ref	235 (32)	1.39 (1.16–1.67)
eGFR 30–20, adj	739	99 (13)	0.76 (0.56–1.04)	405 (55)	ref	235 (32)	1.40 (1.16–1.68)
		53 events		647 events			
eGFR ≤ 20	985	99 (10)	0.61 (0.46–0.81)	886 (90)	ref	NA	NA
eGFR ≤ 20, adj	985	99 (10)	0.73 (0.55–0.97)	886 (90)	ref	NA	NA

Adj = adjusted for age, gender and Charlson Comorbidity Index.

## Discussion

This study includes intensive care patient data from three Swedish University Hospitals and shows that eGFR estimated with cystatin C, alone or in combination with creatinine, was more closely associated with risk of death of all causes as compared to creatinine-based eGFR. More specifically, the sole cystatin C eGFR equation predicted mortality better than the combined equation. The associations were found in the whole sample as well as in the more critically ill subgroup at the general ICU. The underlying mechanisms for these associations are uncertain but may relate to either the a superiority of cystatin C as a GFR-biomarker in this setting or possible non-GFR effects of cystatin C associated with mortality or a combination of the two.

The theory of cystatin C being a more suitable GFR-biomarker than creatinine in critically ill patients arises from the well-known disadvantages of creatinine as a biomarker of GFR. The production rate of creatinine is mainly determined by the patients muscle mass. Generally, the reliability of creatinine as an accurate GFR-biomarker assumes a steady-state production, distribution and clearance of creatinine. Critically ill, bedfast, patients may for several reasons not be in steady-state due to ongoing loss of muscle mass^14^ or altered distribution volume due to fluid accumulation i.e. increased total water volume^15^. Other factors may include impared liver function, low meat intake, trauma or fever. All together these factors may potentially lead to a risk of falsely low creatinine values and hence eGFR overestimation in the critically ill patients^6, 16, 17^. Theoretically, Cystatin C may have advantages over creatinine in estimating eGFR. Cystatin C is produced by all nucleated cells and is not dependent of muscle mass^18^. Further Cystatin C is freely filtered in glomeruli and not affected by malnutrition^19^. One concern is that cystatin C generally may have a high turnover in critical illnesses such as sepsis and/or inflammation causing falsely too high concentrations possibly leading to underestimating eGFR^4, 10, 11^. However, a causal role between inflammation and cystatin C has been under debate and is difficult to establish^20^. Despite the theoretical advantages that cystatin C may have over creatinine as an eGFR-biomarker in critically ill patients, it has not been convincingly shown that cystatin C alone is more accurately related to golden standard measured GFR with iohexol clearance than creatinine alone. A study by Delanaye et al. showed that cystatin C was more closely related to measured GFR than creatinine alone in critically ill patients^21^. Another study showed that combined creatinine-cystatin C eGFR-equations or a mean of cystatin C eGFR and creatinine eGFR show the highest agreement with iohexol clearance in critically ill^17^. This high agreement between the combined equation and measured GFR has also been reported by the CKD-EPI group in patients with chronic kidney disease and in the general population^22, 23^. Thus, these findings do not point out cystatin C alone as the most accurate biomarker for eGFR so this is not the whole explanation for the superior performance of cystatin C in prognosticating mortality.

Temporary acute kidney injury is common in critically ill patients and clearly related to fatal outcome^8, 24^ but according to our data even one single measurement of Cystatin C at admission indicated a long-term mortality risk. Could non-GFR related factors underlie the strong association between cystatin C and long-term mortality? Cystatin C has been correlated with mortality independently of renal function in ICU patients^8^, a finding which is in line with this theory. Another study corroborates with this finding and found that cystatin C was correlated to all-cause mortality despite normal creatinine levels^25^. Traditional cardiovascular risk factors such as diabetes, obesity, smoking, hypertension, insulin resistance and inflammation and also non-traditional risk factors (symmetric dimetylarginine) have been linked to cystatin C^10–13^, and these diseases were highly prevalent as discharge diagnoses in this patient cohort. It cannot be ruled out that the reason why cystatin C predicts mortality better than creatinine is probably, at least in part, due to non-GFR related CVD factors affecting cystatin C. Similar results that the sole cystatin C equation outperformes combined equations or sole creatinine equations for mortality estimation have been shown for other patient groups than critically ill such as unselected patients seeking health care^26^. Further, a meta-analysis of community-based and CKD cohorts by the CKD-prognosis consortium, where cystatin C-based eGFR equations, alone or in combination with creatinine, strengthened the association between eGFR and death at all different levels of eGFR. However, despite the consistent superiority of cystatin C based eGFR over creatinine based GFR in previous studies, a recent large scale Mendelian randomization analyses, predominantly based on community based studies, did not support a causal role of circulating cystatin C in the development of cardiovascular disease^27^. Additional studies are warranted to provide further insights into the underlying mechanisms of these associations.

All analyses were performed at accredited University Hospital laboratories with methods traceble to the international standard calibration which is a strength in this study. One laboratory changed the creatinine method from Jaffe to enzymatic during the study period. Jaffe methods are known to potentially overestimate creatinine, however at the time of the study inclusion the Jaffe methods at Swedish hospital laboratories were recalibrated to harmonize with enzymatic methods^28^. Hence, the creatinine methods used in this study are sufficiently comparable for the included subjects and for the study aim. There were no loss to follow up due to the high quality of Swedish registry data^29^. We are not aware of any larger study comparing creatinine and cystatin C for risk prediction purposes in critically ill patients and we believe that since three large University Hospitals contributed with data the generalizability to critically ill patients in general increase. We did not include the factor for African-Americans in the formulas in this study based on the knowledge that study participants are predominantly Caucasian. This should not have biased our results but we acknowledge however that the study results may have limited generalizability to other ethnicities than Caucasian. Charlson comorbidity index only records prior hospital care, comorbidity treated in primary care may therefore have been missed which is a disadvantage. Given the possible non-GFR related connection between inflammation and cystatin C that has been proposed it is a disadvantage that the study lacks an inflammatory variable as a covariate in the models. Concerns have been raised that the net reclassification index may provide false positive findings during certain circumstances^30^, however, given the consistency of the superiority of cystatin C based GFR throughout the full range of eGFR provide support for the validity of our findings.

A sole cystatin C equation for eGFR consistently predicted mortality risk better as compared to the sole creatinine-based equation or the combined creatinine/cystatin C equation in patients at intensive care units. Thus, our data favours the use of the sole cystatin C equation rather than the combined cystatin C-creatinine equation when estimating GFR for risk prediction purposes in critically ill patients.

## Methods

### Study population

The retrospective observational study is based on simultaneous measurements of plasma creatinine and cystatin C on adult patients at admission to intensive care units at Uppsala, Karolinska and Lund University Hospitals, Sweden, from 2004 to 2015. These hospitals perform the vast majority of all cystatin C analyses in Sweden. The patients’ samples were analysed at Uppsala, Karolinska and Lund University Hospital Laboratories, respectively, on the same fresh plasma sample tube. Valid quantitative result of creatinine, cystatin C, age and gender were extracted from the laboratory information systems. All Swedish citizens and those with residence permit have personal identity numbers. Only patients with a complete personal identity number, 16 years of age and older, were included. If participants had more than one measurement only the first measurement was included in the study. In total, 22,488 unique patients with simultaneous measurements of plasma creatinine and cystatin C were included. Swedish Ethical Review Authority in Uppsala approved the research protocol, Dnr 2013/441. All methods were carried out in accordance with relevant guidelines and regulations and reporting followed the STROBE Statement. Informed consent from subjects was waived by the Swedish Ethical Review Authority in Uppsala since only anonymised registry data was analysed.

### Measurement of creatinine, cystatin C and estimation of glomerular filtration rate (eGFR)

Plasma creatinine (µmol/L) was analysed with a modified kinetic Jaffe method 2004–2008 and an enzymatic method 2009–2015 on Architect ci8200 (Abbott Laboratories, Abbott Park, Ill., USA) in Uppsala, modified kinetic Jaffe method on UniCel DXC800 (Beckman Coulter, Brea, CA) at Karolinska and an enzymatic method on Roche Cobas c501 (Roche Diagnostics, Rotkreuz, Switzerland) in Lund. All methods were IDMS calibrated by the manufacturer and all three hospital laboratories were accredited. eGFR_Cr_, in mL/min/1.73 m^2^, was estimated using The Chronic Kidney Disease Epidemiology Collaboration (CKD-EPI) creatinine equation from 2009^31^. Plasma cystatin C was analysed with reagents from Dade Behring on a BN ProSpec analyzer (Siemens Healthcare Diagnostics) at Uppsala and Karolinska University Hospitals until 2007 and thereafter with an assay from Gentian (Gentian, Moss, Norway), traceable to the international calibrator ERM-DA471/IFCC, on Architect ci8200 in Uppsala^32, 33^, and on UniCel DXC800 at Karolinska. Cystatin C was analysed with reagents from Roche, traceable to the international calibrator ERM-DA471/IFCC^34^, on Roche Cobas c501 in Lund. eGFR_Cyst_ in mL/min/1.73 m^2^ was calculated from plasma cystatin C using CAPA^35^. The international IFCC-equation Caucasian, Asian, Pediatric, and Adult cohorts (CAPA) is practically assay-independent since ERM-DA471/IFCC calibrated cystatin C-assays from 7 diagnostic companies are used. Further, the equation is based on Caucasian and Asian, both pediatric and adult cohorts. eGFR_Comb_ in mL/min/1.73m^2^ was calculated using the CKD-EPI combined creatinine/cystatin C equation (2012)^22^.

### Comorbidity data and endpoint definition

Comorbidity data from 2004 and forward for calculating the Charlson Comorbidity Index (CCI) i.e. in hospital care prior to the study intensive unit care was collected from the National Patient Register that collects data from all in-patient hospital visits in Sweden. CCI is described in detail in Quan et al.^36^. In brief, the CCI categorizes comorbidities of patients based on the ICD-10 diagnosis codes and sorted into categories. Included categories were myocardial infarction, congestive heart failure, peripheral vascular disease, cerebrovascular disease, chronic pulmonary disease, rheumatic disease, lever disease, diabetes with complications, diabetes without complication, hemiplegia or paraplegia, renal disease and HIV/AIDS. Each comorbidity category weights from 1 to 6 (based on mortality risk) and the sum of all the weights is the comorbidity score for a patient. A score of zero indicates no comorbidities. A higher score is associated with a higher mortality risk. Three thousand five hundred and ten patients (16%) had no prior hospital care recorded in the National Patient Register. The endpoint mortality was defined using the Swedish Cause of Death Register for all participants and there was no loss to follow up. Both registers are administered by the Swedish National Board of Health and Welfare and records data for all Swedish residents.

### Statistics

The associations of eGFR_Cr_, eGFR_Cyst_ and eGFR_Comb_ and mortality, respectively, were analysed in Cox proportional hazard regression models. The univariate hazard ratio was computed for each 1 ml/min/1.73 m^2^ of eGFR from 15 to 120 using a reference point at 95 ml/min/1.73 m^2^^37^ and shown as regression spline curves. The Harrell´s C statistics^38^, 95% confidence intervals and p-values were calculated using the “somersd” package with the “lincom” command by splitting the study population randomly into a training set and a test set. We also divided the participants into risk categories according to the European Society of Cardiology (ESC) clinical cardiovascular prevention guidelines^39^ and the clinical decision limit for dialysis in the intensive care unit 20 ml/min/1.73 m^2^. Thus, the variables were entered into the models in the eGFR categories > 60, 60–30, 30–20 and < 20 mL/min/1.73 m^2^. The study population were classified to an eGFR category by the creatinine equation and the combined equation and proportion of participants who were reclassified to a higher or lower eGFR category by the cystatin C CAPA equation was assessed for mortality risk compared to the participants not reclassified using Cox proportional hazards models. Models were adjusted for the potential confounding variables age, gender and CCI since the patients who reclassified to a higher risk category with cystatin C were generally older, of male gender and had higher CCI.

Overall improvement in reclassification based the eGFR categories > 60, 60–30, 30–20 and < 20 mL/min/1.73 m^2^ was evaluated using net reclassification improvement (NRI) according to Pencina et al.^40^ *P* values < 0.05 were regarded as statistically significant. Calculations were performed with Stata 13 (Stata Corp., College Station, TX, USA).

## Data Availability

The datasets generated during and/or analysed during the current study are not publicly available due to the reason that the datasets used contain information that potentially could identify individual patients. Authors are willing to share their data on reasonable request and after case-by-case assessment of such request by a local ethics committee.

## Abbreviations

CKD: Chronic kidney disease
eGFR: Estimated glomerular filtration rate
HR: Hazard ratio
IFCC: International federation of clinical chemistry
NRI: Net reclassification improvement
